# β2 Integrins Differentially Regulate Macrophage Polarization, Inflammatory Signaling, and Function: A Prominent Role for CD11d

**DOI:** 10.21203/rs.3.rs-11007877/v1

**Published:** 2026-09-28

**Authors:** Fahd Alhamdan, Ganchimeg Bayarsaikhan, Sophia Koutsogiannaki, Koichi Yuki

**Affiliations:** Boston Children's Hospital; Ragon Institute of MGH, MIT and Harvard; Boston Children's Hospital; Boston Children's Hospital

**Keywords:** β2 integrins, macrophages, M1, proinflammatory

## Abstract

β2 integrins are leukocyte-specific adhesion receptors that regulate immune cell trafficking and outside-in signaling; however, the individual contributions of their four α subunits, CD11a, CD11b, CD11c, and CD11d, to macrophage activation remain incompletely understood. Here, we investigated the expression dynamics and functional roles of β2 integrins during macrophage polarization by integrating bulk and single-cell RNA-sequencing datasets with transcriptomic, pathway, and functional analyses of individual β2-integrin-deficient macrophages. The four β2-integrin subunits displayed distinct temporal expression patterns during lipopolysaccharide (LPS)-induced macrophage activation and differential expression across M0, M1, and M2 macrophage states. Deletion of individual β2 integrins generally enhanced the M1 transcriptional signature but produced distinct cytokine, chemokine, and functional phenotypes. CD11d deficiency induced one of the most pronounced transcriptional alterations, characterized by increased TNF, interferon, IL-10, JAK-STAT, p53, TGFβ, and WNT signaling, accompanied by reduced hypoxia, MAPK, and PI3K pathway activity. CD11b deficiency preferentially enhanced NF-κB, TNFα, and VEGF signaling while reducing phagocytosis. In contrast, CD11a and CD11c deficiency showed partially convergent transcriptional and MAPK-associated signaling profiles but distinct functional consequences. Analysis of inflammatory and functional signatures further demonstrated integrin-specific regulation of phagocytosis, macrophage activation, differentiation, chemotaxis, and MHC class II-associated programs. Moreover, deletion of individual β2-integrin subunits altered the expression of the remaining family members, revealing a dynamic network of compensatory and antagonistic interactions that became more complex following LPS stimulation. CD11d deficiency was associated with increased inflammatory infiltration during LPS-induced acute lung injury, providing an *in vivo* correlate of its enhanced inflammatory macrophage phenotype. Together these findings demonstrate that β2 integrins differentially regulate macrophage polarization, signaling, and effector functions rather than acting as functionally redundant adhesion receptors.

## Introduction

Macrophages are highly plastic innate immune cells that play central roles in tissue homeostasis, host defense, inflammation, and tissue repair^1^. In response to environmental cues, macrophages undergo substantial transcriptional and functional reprogramming, enabling them to adopt diverse activation states. Although the classical M1/M2 framework represents a simplified model of macrophage polarization, it remains useful for describing the contrasting proinflammatory programs induced by stimuli such as lipopolysaccharide (LPS) and the alternative activation programs associated with cytokines including interleukin-4 (IL-4)^2,3^. LPS activation induces macrophage inflammatory responses through Toll-like receptor signaling and downstream pathways such as NF-κB and MAPK, resulting in the production of cytokines, chemokines, interferon-responsive genes, and other mediators that coordinate innate immune responses^4,5^. However, excessive or dysregulated macrophage activation can contribute to inflammatory tissue injury, highlighting the importance of mechanisms that fine-tune macrophage signaling and function.

Integrins are heterodimeric cell-surface receptors that provide an important interface between cells and their extracellular environment^6^. In addition to mediating cell adhesion and migration, integrins regulate intracellular signaling through bidirectional communication across the plasma membrane^7^. While inside-out signaling regulates integrin activation and ligand-binding affinity, outside-in signaling initiated following ligand engagement influences cytoskeletal organization, survival, migration, proliferation, and inflammatory gene expression^8^. These signaling properties place integrins in a strategic position to coordinate environmental sensing with immune cell activation.

The β2-integrin family is expressed predominantly by leukocytes and consists of a common β2 subunit, CD18, paired with one of four α subunits: CD11a (αL; encoded by *Itgal*), CD11b (αM; *Itgam*), CD11c (αX; *Itgax*), or CD11d (αD; *Itgad*)^9,10^. Although these integrins share the common CD18 chain, they differ in their ligand-binding properties, cellular distribution, and biological functions. CD11a/CD18, also known as LFA-1, is widely expressed on leukocytes and is particularly important for immune-cell adhesion and interactions through intercellular adhesion molecules (ICAMs)^11^. CD11b/CD18, or Mac-1, is highly expressed by myeloid cells and has been extensively implicated in leukocyte adhesion, migration, phagocytosis, complement recognition, and inflammatory signaling^12,13^. CD11c/CD18 is expressed by dendritic cells and macrophages and has been associated with antigen presentation, cellular activation, and immune regulation^14,15^. In contrast, CD11d/CD18 remains comparatively less well characterized. CD11d is expressed in several leukocyte populations, including macrophages, and has been implicated in leukocyte adhesion, migration, and inflammatory responses in different disease settings^16,17^.

Despite extensive evidence that individual β2 integrins contribute to leukocyte trafficking and immune function, considerably less is known about how the four β2-integrin α subunits collectively regulate macrophage activation and polarization. Because these receptors share the CD18 β chain and can recognize overlapping extracellular ligands, functional redundancy or compensatory interactions may exist among family members.

In this work, we characterize the temporal and polarization-associated expression dynamics of all four β2-integrin subunits in macrophages, define the transcriptional and signaling consequences of individual subunit deletion under baseline and LPS-stimulated conditions, and identify CD11d as a subunit whose loss produces a disproportionately strong shift toward M1-like, TNF-driven macrophage activation, a phenotype we further validate at the tissue level using an in vivo LPS lung injury model.

## Results

### β2 integrins display distinct temporal and polarization-associated expression patterns in macrophages

To investigate the temporal expression dynamics of the four β2 integrin subunits, Itgal (CD11a), Itgam (CD11b), Itgax (CD11c), and Itgad (CD11d), during macrophage activation, we reanalyzed publicly available bulk RNA-sequencing data (GSE103958) from bone marrow-derived macrophages (BMDMs) stimulated with LPS to induce an M1-like proinflammatory phenotype over a 24-h time course^18^ (Fig. 1a). The four β2 integrins exhibited distinct and temporally regulated expression patterns. Itgal showed an early increase in expression, becoming elevated as early as 4 h after LPS stimulation, suggesting that CD11a may participate in the early transcriptional response to inflammatory activation. In contrast, Itgam expression was significantly reduced at 4 h and subsequently returned toward baseline by 24 h, indicating a transient suppression during the early phase of M1 polarization. Itgax exhibited a progressive and marked reduction throughout the 24-h stimulation period. Itgad showed a modest early increase at 4 h, followed by a continuous decline toward 24 h. These findings demonstrate that the four β2 integrin subunits are not coordinately regulated during macrophage activation, but instead display distinct temporal responses to LPS stimulation, suggesting that individual β2 integrins may have specialized roles during different stages of macrophage activation.

We next sought to determine whether these expression patterns were associated with specific macrophage polarization states. To this end, we reanalyzed publicly available single-cell RNA-sequencing data (GSE161125) comprising M0 (baseline/controls), M1 (proinflammatory/LPS-stimulated), and M2 (anti-inflammatory/IL-4-stimulated) macrophages^19^ (Fig. 1b). We first examined the distribution of Itgal, Itgam, Itgax, and Itgad across individual macrophages and quantified the proportion of integrin-positive cells in each polarization state (Fig. 1c). Itgal^+^ macrophages increased in frequency in the M1 state, accompanied by increased Itgal expression, whereas Itgam^+^ macrophages decreased in frequency during M1 polarization. Indicating that the increased Itgal and Itgam signals during M1 polarization appear to be at least partly associated with a change in proportion of integrin-positive macrophages, whereas the relatively modest changes in the frequency of Itgax^+^ and Itgad^+^ cells suggest that their transcriptional regulation may contribute more substantially to their polarization-associated expression patterns.

To further characterize expression intensity at the single-cell level, we generated density plots of integrin expression across macrophage polarization states (Fig. 1d). Itgal and Itgad showed higher expression densities in M1 macrophages, indicating preferential expression of these integrins within the proinflammatory state. Itgax was most prominently expressed in M2 macrophages. Itgam displayed the highest expression in M0 macrophages, followed by M2 and M1 macrophages. Thus, although all four β2 integrins form the same integrin sub-family, their expression is differentially regulated according to macrophage activation state.

### β2-Integrin Deficiency Differentially Regulates the Macrophage M1 Transcriptional Program

To further define the contribution of individual β2-integrin subunits to macrophage inflammatory activation, we performed RNA-sequencing analysis of WT and β2-integrin-deficient macrophages under baseline and LPS-induced M1 conditions. Principal component analysis (PCA) revealed distinct transcriptional profiles among the different β2-integrin-deficient macrophages at both baseline and M1 states (Fig. 2a). Under baseline conditions, Itgam^−/−^ and Itgad^−/−^ macrophages clustered closely with WT macrophages, suggesting that CD11b and CD11d have relatively limited effects on the global macrophage transcriptome under steady-state conditions. While Itgal^−/−^ and Itgax^−/−^ macrophages were far away at baseline, they clustered closely together following LPS stimulation, suggesting shared transcriptional responses and potential functional overlap between CD11a and CD11c during M1 polarization.

We next assessed the M1 signature score, calculated based on genes upregulated during M1 compared with M0 polarization, to determine whether β2-integrin deficiency altered the extent of inflammatory macrophage activation (Fig. 2b). Loss of individual β2-integrin subunits generally increased the M1 signature score, suggesting that β2-integrin deficiency promotes a more pronounced M1-like transcriptional state following LPS stimulation. The increase was most evident across the Itgal^−/−^, Itgam^−/−^, and Itgad^−/−^ macrophages, whereas the increase in Itgax^−/−^ macrophages did not reach statistical significance. This latter finding is consistent with the preferential expression of Itgax in M2 macrophages.

We next examined the expression of key inflammatory cytokine and chemokine genes associated with M1 macrophage activation, including Cxcl1, Ifng, Il10, Il12a, Il12b, Il1b, Il6, and Tnf (Fig. 2c). Cxcl1 expression was markedly reduced in Itgax^−/−^ and Itgad^−/−^ macrophages, whereas Ifng expression was not significantly affected by deletion of any β2-integrin subunit. Il10 expression was increased in Itgal^−/−^, Itgam^−/−^, and Itgax^−/−^ macrophages, with the strongest increase observed following Itgal deletion. Similarly, Il12a expression was increased in Itgal^−/−^, Itgam^−/−^, and particularly Itgax^−/−^ macrophages. In contrast, Il12b expression was reduced in Itgal^−/−^ and Itgax^−/−^ macrophages but increased in Itgam^−/−^ and Itgad^−/−^ macrophages. Expression of Il1b and Il6 was significantly reduced in both Itgam^−/−^ and Itgad^−/−^ macrophages, whereas Tnf expression was increased in these genotypes, with the most pronounced increase observed in Itgad^−/−^ macrophages. These findings demonstrate that β2-integrin deficiency does not uniformly suppress or enhance the inflammatory response but instead produces distinct, integrin-specific changes in the M1 transcriptional program.

To further define the molecular pathways affected by individual β2-integrin subunits, we examined the expression of genes representing major inflammatory and immune signaling pathways in β2-integrin-deficient macrophages stimulated with LPS (Fig. 2d). Within the TLR signaling pathway, Itgad^−/−^ macrophages showed increased expression of several TLR-associated genes, including Tlr3, Tlr9, Table 2, and Table 1, together with increased Rel expression. In contrast, Itgam^−/−^ macrophages showed relatively limited changes in this pathway. Cytokine and chemokine-associated genes displayed broader alterations across the β2-integrin knockouts. Several chemokines, including Cxcl9, Ccl8, Ccl7, Cxcl11, and Cxcl10, were increased in multiple knockout conditions, whereas Cxcl1 and Cxcl3 showed more selective changes. Multiple interferon-stimulated genes, including Ifi207, Ifi205, Ifi204, Oas2, Ifi209, Ifit2, Mx2, Mx1, Ifi214, Ifi211, Ifit3, Oasl1, Ifi206, and Ifitm family members, were upregulated across the β2-integrin-deficient macrophages, particularly Itgad^−/−^ macrophages.

Complement-associated genes, including C2, C1qa, C4b, C1qc, C1qb, C3, and C5ar1, were also differentially expressed across the knockout groups, with particularly evident changes in Itgax^−/−^ and Itgad^−/−^ macrophages.

Itgam^−/−^ macrophages showed marked reduction of Itgam and alterations in Cx3cr1, Itgal, Itgax, and other adhesion-related genes. These findings further support a central role for β2-integrins in coordinating macrophage adhesion and migratory programs.

Inflammasome-associated genes showed comparatively restricted changes. Casp12, Nlrp3, Aim2, P2rx7, Mefv, Casp4, Nek7, and Txnip were differentially expressed in selected knockout conditions, with several genes showing increased expression in Itgal^−/−^ or Itgad^−/−^ macrophages. In contrast, the expression changes within this pathway were less extensive than those observed for cytokine/chemokine and type I interferon-associated genes.

Antigen-presentation genes, including Cd74, H2-Aa, H2-Eb1, Ciita, Cd80, Cd86, Tap1, and Cd40, were differentially affected, with several genes showing reduced expression in specific knockout conditions and increased expression particularly in Itgad−/− macrophages.

### β2-Integrin Deficiency Differentially Modulates Macrophage Signaling Pathways

To evaluate how loss of individual β2-integrin subunits reshapes intracellular signaling, we applied PROGENy to infer pathway activity scores and compared their distributions across WT and Itgal^−/−^ Itgam^−/−^, Itgax^−/−^, and Itgad^−/−^ macrophages following 12 hours of LPS stimulation (Fig. 3).

Itgad^−/−^ macrophages showed the most distinct signaling signature among the knockouts, with pronounced rightward shifts toward higher pathway activity in JAK-STAT, p53, TGFβ, TNFα, and WNT, alongside a marked leftward shift toward lower Hypoxia, MAPK, and PI3K activity relative to WT. Itgam^−/−^ macrophages displayed a convergent but distinct profile, with elevated NFκB, p53, TNFα, and VEGF activity, while EGFR, MAPK, and Trail scores remained close to baseline.

In contrast, Itgad^−/−^ and Itgax^−/−^ macrophages showed a shared shift toward increased MAPK activity, with Itgal^−/−^ additionally showing a sharp, narrow peak in EGFR activity not seen in other genotypes. Both Itgal^−/−^ and Itgax^−/−^ also trended toward modestly elevated hypoxia and TNFα activity compared with WT.

WT macrophages were characterized by tightly distributed, low-magnitude activity across most pathways, most notably showing distinct peaks of low hypoxia, low JAK-STAT, low NFκB, low TNFα, and low VEGF activity, as well as a uniquely sharp Trail and Estrogen peak near baseline that was broader or absent in the knockout groups. These data indicate that individual β2-integrin subunits differentially rewire intracellular signaling downstream of receptor engagement, with Itgad loss producing the broadest divergence from WT, engaging pro-inflammatory (TNFα, JAK-STAT) and stress-response (p53, TGFβ) pathways while suppressing hypoxia and MAPK signaling, whereas Itgam loss more selectively amplifies NFκB and VEGF activity, and Itgal/Itgax loss converges on enhanced MAPK signaling.

PROGENy pathway activity scores for WT, Itgal−/−, Itgam−/−, Itgax−/−, and Itgad−/− bone marrow-derived macrophages, showing the activity profiles of major signaling pathways, including androgen, EGFR, estrogen, hypoxia, JAK–STAT, MAPK, NFκB, p53, PI3K, TGFβ, TNFα, TRAIL, VEGF, and WNT signaling.

### β2-Integrin Deficiency Dictates Cytokine Signaling and Macrophage Functions

To further determine whether individual β2-integrin subunits regulate specific macrophage signaling and functional programs, we compared cytokine signaling and functional signature scores across WT, Itgal^−/−^, Itgam^−/−^, Itgax^−/−^, and Itgad^−/−^ macrophages (Fig. 4a) (**Supplementary Table 1**). Among the inflammatory signaling pathways, CD11d deficiency produced one of the most pronounced phenotypes. Itgad^−/−^ macrophages showed markedly increased TNF and IFN signaling, accompanied by increased IL-10 signaling, while IL-1 and IL-17 signaling were significantly reduced. The strong increase in TNF signaling in Itgad^−/−^ macrophages was consistent with the increased Tnf expression observed in these cells. CD11b deficiency also substantially altered inflammatory signaling, with increased IL-12, IFN, and IL-10 signaling. In contrast, CD11b-deficient macrophages showed reduced phagocytosis (Fig. 4b) (**Supplementary Table 2**), indicating that enhanced inflammatory signaling was not accompanied by a generalized increase in macrophage effector function.

CD11a deficiency was characterized by a broader increase in inflammatory signaling (Fig. 4a). Itgal^−/−^ macrophages exhibited increased IL-6, IL-1, IFN, IL-17, and IL-10 signaling compared with WT macrophages. Functionally, these cells also showed increased phagocytosis and macrophage activation (Fig. 4b), suggesting that loss of CD11a is associated with a more activated and functionally responsive macrophage state. CD11c deficiency displayed a comparatively selective phenotype, with particularly strong enhancement of IL-1 signaling and increased phagocytosis and differentiation scores, whereas IL-12 and IL-17 signaling remained relatively similar to WT (Fig. 4a–b).

The functional signatures further demonstrated that individual β2-integrins have distinct effects on macrophage behavior. Itgam^−/−^ macrophages showed the lowest phagocytosis score but increased MHC class II expression and chemotaxis (Fig. 4b), suggesting a shift away from phagocytic activity toward antigen-presentation and migratory programs. In contrast, Itgal^−/−^ macrophages showed increased phagocytosis and activation, while Itgad^−/−^ macrophages displayed increased differentiation, MHC class II expression, and chemotaxis. Differentiation was broadly increased following β2-integrin deficiency, with the strongest phenotype observed in Itgad^−/−^ macrophages. Notably, foaming scores were not significantly altered by deletion of any β2-integrin subunit, indicating that this functional program was relatively preserved despite substantial changes in inflammatory signaling and other macrophage functions.

### β2-Integrin Subunits Form a Dynamic and Interconnected Regulatory Network

To investigate the regulatory relationships among the four β2-integrin subunits, we examined how deletion of each individual subunit affected the expression of the remaining integrins under control and LPS-stimulated conditions (Fig. 5a–d). To quantify these relationships, we calculated the log2 fold-change in each β2-integrin following deletion of the other subunits (KO/WT; Fig. 5a). The most prominent positive effects included increased Itgad expression following Itgam and Itgal deletions, particularly under LPS stimulation. Conversely, deletion of Itgad produced marked reductions in Itgam and Itgal expression, while Itgax deficiency was associated with reduced Itgam expression, suggesting that certain β2-integrin subunits may compensate for the loss of another, whereas others may be coordinately regulated or exhibit antagonistic relationships. (Fig. 5b–c) Heatmaps analysis further demonstrated the same previous findings with LPS.

We next integrated these pairwise effects into a network model to distinguish compensatory from antagonistic relationships (Fig. 5c). At baseline, the network was dominated by compensatory interactions. Following LPS stimulation, the network became more complex, with both compensatory and antagonistic interactions emerging. In particular, Itgam and Itgad occupied prominent positions within the network, showing multiple connections with the other β2-integrins. Itgal and Itgax also participated in reciprocal interactions, although their connectivity differed between control and LPS conditions.

### CD11d deficiency enhanced inflammatory infiltration during acute lung injury

As previously shown, deficiency of β2-integrin subunits increased the M1 signature score, with the highest score observed under CD11d-deficient conditions, accompanied by significantly increased *Tnf* expression levels and TNF signaling score. Consistent with this, histological examination of lung sections following LPS instillation revealed a more pronounced inflammatory infiltrate in CD11d-deficient mice compared with WT. WT lungs showed relatively preserved alveolar architecture with modest, patchy septal cellularity (Fig. 6), whereas CD11d-KO lungs exhibited denser interstitial and intra-alveolar cellular infiltration accompanied by increased capillary congestion (Fig. 6), providing a morphological correlate of the enhanced M1-like, TNF-driven macrophage activation observed at the transcriptional level, indicating that the combined increase in M1 signature and TNF elevation results in both an intensified inflammatory macrophage transcriptional program and a corresponding increase in inflammatory cell infiltration during acute LPS-induced lung injury.

## Discussion

Our study provides a comparative analysis of CD11a, CD11b, CD11c, and CD11d during macrophage activation and demonstrates that, despite sharing the common CD18 subunit, these β2 integrins are differentially regulated and exert distinct effects on macrophage inflammatory programs. The divergent temporal expression patterns observed during LPS stimulation, together with their differential distribution across M0, M1, and M2 macrophages, support the concept that β2 integrins are not functionally redundant but contribute to distinct aspects of macrophage activation and environmental sensing^9,20^. β2 integrins regulate not only leukocyte adhesion and migration but also outside-in signaling, phagocytosis, and inflammatory responses, providing a mechanistic basis for the broad transcriptional changes observed following deletion of individual subunits^21^.

A major finding from our work was that deficiency of individual β2 integrins generally enhanced the M1 transcriptional signature but produced distinct cytokine and signaling phenotypes. This suggests that β2 integrins may function as context-dependent regulators that fine-tune, rather than uniformly promote or suppress, macrophage inflammation. Consistent with this, β2 integrins have been shown to regulate different TLRs in distinct ways, with loss of β2 integrins causing TLR hyperresponsiveness and altered downstream signaling, including changes in MyD88-dependent NF-κB and p38 MAPK activation^8,22^. The phenotype of CD11b-deficient macrophages, characterized by altered inflammatory signaling but reduced phagocytosis, is consistent with the established role of CD11b/CD18 as an important phagocytic and complement receptor. Previous studies have also shown that CD11b can either enhance or restrain TLR-mediated responses depending on the cellular context, highlighting the complexity of β2-integrin signaling^21,23,24^.

CD11d deficiency produced one of the most pronounced phenotypes, with enhanced M1 signatures, increased Tnf expression and TNF signaling, and broad alterations in TLR-, interferon-, complement-, and antigen-presentation-associated programs. These changes were accompanied by substantial remodeling of inferred intracellular signaling pathways. Although CD11d remains the least characterized member of the β2-integrin family, previous studies have demonstrated its expression on macrophages and its ability to mediate outside-in signaling and inflammatory gene expression^25,26^. CD11d is also upregulated on inflammatory macrophages and can promote macrophage retention at sites of inflammation, suggesting that its effects may extend beyond adhesion to the regulation of macrophage activation and inflammatory persistence^25,27^.

The distinct phenotypes of CD11a- and CD11c-deficient macrophages further support nonredundant functions within the β2-integrin family. CD11a deficiency was associated with increased inflammatory signaling and phagocytosis, whereas CD11c deficiency preferentially affected IL-1 signaling, phagocytosis, and differentiation. The close transcriptional clustering of CD11a- and CD11c-deficient macrophages following LPS stimulation, together with their shared increase in MAPK activity, suggests partial convergence of their downstream signaling programs. However, the differences in their functional signatures indicate that common signaling changes do not necessarily produce identical macrophage phenotypes.

An additional important finding was the dynamic regulatory network among the four integrins. Deletion of one β2-integrin subunit altered the expression of others, and these relationships became more complex following LPS stimulation. In particular, the reciprocal effects involving Itgam and Itgad suggest the presence of compensatory or antagonistic mechanisms within the β2-integrin family. Because these receptors share CD18 but differ in ligand specificity and signaling capacity, changes in one integrin may influence receptor composition, downstream signaling, or transcriptional feedback regulating the remaining family members^11,28^.

Several limitations should be considered. The M1/M2 framework represents a simplified model of macrophage activation, and the transcriptomic findings require validation at the protein and functional levels. In addition, global integrin deficiency may induce developmental or compensatory effects. Future studies using conditional deletion models, integrin activation or blocking approaches, and direct measurements of outside-in signaling will be important to define the underlying mechanisms.

Overall, our findings demonstrate that CD11a, CD11b, CD11c, and CD11d differentially regulate macrophage activation, signaling, and function. Rather than acting as a redundant group of adhesion receptors, β2 integrins appear to form a dynamic regulatory network that shapes macrophage responses during inflammation. Notably, CD11d deficiency produced a particularly broad inflammatory and transcriptional phenotype and was associated with increased inflammatory infiltration during acute lung injury, identifying CD11d as a potentially important regulator of macrophage-mediated inflammation.

## Methods

### Mice

Wild type (WT), CD11a (αL) KO, CD11b (αM) KO, and CD11d (αD) KO mice were obtained from Jackson Laboratory (Bar Harbor, Maine, USA). CD11c KO mice were kindly given by Dr. Ballantyne (Bayler University). They were housed under specific pathogen-free condition, with 12-hour light and dark cycles. All animal protocols were approved by the Institutional Animal Care and Use Committee (IACUC) at Boston Children’s Hospital.

### Bone marrow macrophage isolation and LPS stimulation

Bone marrow cells were collected from the femur of WT, CD11a mice, CD11b mice, CD11c KO mice and cultured for 7 days in the presence of M-CSF for macrophage differentiation. Then macrophages were stimulated with LPS (1 μg/mL) for 6 hours before collection.

### RNA extraction and library preparation

Macrophages were subjected to RNA extraction using Qiagen RNeasy kit. RNA purity was monitored via a NanoDrop spectrophotometer (Thermo Fisher Scientific) and integrity were quantified using the Qubit RNA Assay Kit in a Qubit 2.0 Fluorometer (Life Technologies) and the RNA Nano 6000 Assay Kit on the Agilent Bioanalyzer 2100 system (Agilent Technologies), respectively. Only samples with an RNA Integrity Number (RIN) > 7.0 were used for downstream library RNA library construction was performed by Novogene (Novogene Co., Ltd. /Sacramento) using a total of 1 μg of RNA per sample as input. Polyadenylated mRNA was enriched using oligo(dT)-attached magnetic beads. Ribosomal RNA was removed according to the manufacturer’s instructions. First-strand cDNA was synthesized using random hexamer primers and M-MuLV Reverse Transcriptase. Second-strand cDNA synthesis was subsequently performed using DNA Polymerase I and RNase H. Remaining overhangs were converted into blunt ends via exonuclease/polymerase activities. Following 3' adenylation, NEBNext Adaptors with hairpin loop structures were ligated to the cDNA fragments. To select cDNA fragments of preferentially 250–300 bp in length, library fragments were purified with the AMPure XP system (Beckman Coulter). PCR was performed using Phusion High-Fidelity DNA Polymerase, Universal PCR primers, and Index (X) Primer. PCR products were purified with AMPure XP beads, and library quality was assessed on the Agilent Bioanalyzer 2100 system. Clustering of the index-coded samples was performed on a cBot Cluster Generation System (or equivalent onboard flow-cell clustering). After cluster generation, libraries were sequenced on an NovaSeq X Plus platform to generate 150 bp paired-end reads.

### Bulk RNA-Seq data analysis

RNA-seq data were processed using the Galaxy platform. Raw sequencing read quality was assessed with FastQC (v0.72) as we previously performed^29^, and reads were aligned to the mouse reference genome (Mus musculus, mm10/GRCm38) using RNA STAR (v2.7.8a) with default parameters. Uniquely mapped reads were assigned to genes and quantified using featureCounts (v2.0.1). Differential gene expression analysis was performed using DESeq2 (v1.40.6). Genes with an adjusted P value (Benjamini–Hochberg) < 0.05 and an absolute log2fold change > 0.57 were considered significantly differentially expressed. Variance-stabilizing transformation (VST) was applied to raw count data using DESeq2 for downstream analyses. Functional enrichment analysis of differentially expressed genes was performed using the clusterProfiler package (v4.12.6) with Gene Ontology (Biological Process) and Reactome pathway annotations. Pathway activity was inferred using the progeny package (v1.26.0). Functional modules were performed by calculating the average expression of all sample and followed by Z scores calculation.

### Single-cell processing, clustering, and downstream analysis

Single-sample .h5 files were processed using the Seurat package (v5.2.1) as we previously performed^30^. Cells expressing fewer than 200 detected genes and genes detected in fewer than three cells were excluded during quality control. Cells with > 10% mitochondrial transcripts were also removed. Following metadata annotation, individual Seurat objects were merged, normalized, and scaled for downstream analyses. Dimensionality reduction and unsupervised clustering were performed using the standard Seurat workflow with a clustering resolution of 0.3 to identify major cell populations. To minimize technical batch effects, data integration was performed using the Harmony package (v1.2.3). Density plots were generated using the Nebulosa package (v1.14.0). Gene module scores were calculated using the AddModuleScore function in the Seurat package, and the percentage of cells positive for each gene module was subsequently determined.

### Intranasal LPS stimulation

WT and αD KO mice were anesthetized using ketamine and xylazine. 10 μg of LPS was given intranasally and mice were euthanized at 6 hours after instillation. Lungs were harvested and fixed in 4% paraformaldehyde (PFA) before embedding in paraffin. Tissues were sectioned for hematoxylin and eosin (HE) staining.

### Statistical analysis

Statistical comparisons between two groups were performed using the Wilcoxon rank-sum test or two-tailed Student’s t-test as appropriate. Multiple group comparisons were performed using one-way ANOVA with Bonferroni post-hoc correction. Pearson correlation coefficients were calculated for regression analyses. Significance thresholds: *p < 0.05, **p < 0.01, ***p < 0.001, ****p < 0.0001. All statistical analyses were performed in R (v2024.04.2 + 764).

## Supplementary Material

Supplementary Files

This is a list of supplementary files associated with this preprint. Click to download.

- SupplementaryTable2B2revised.xlsx
- SupplementaryTable1B2revised.xlsx

## Figures and Tables

**Figure 1:**
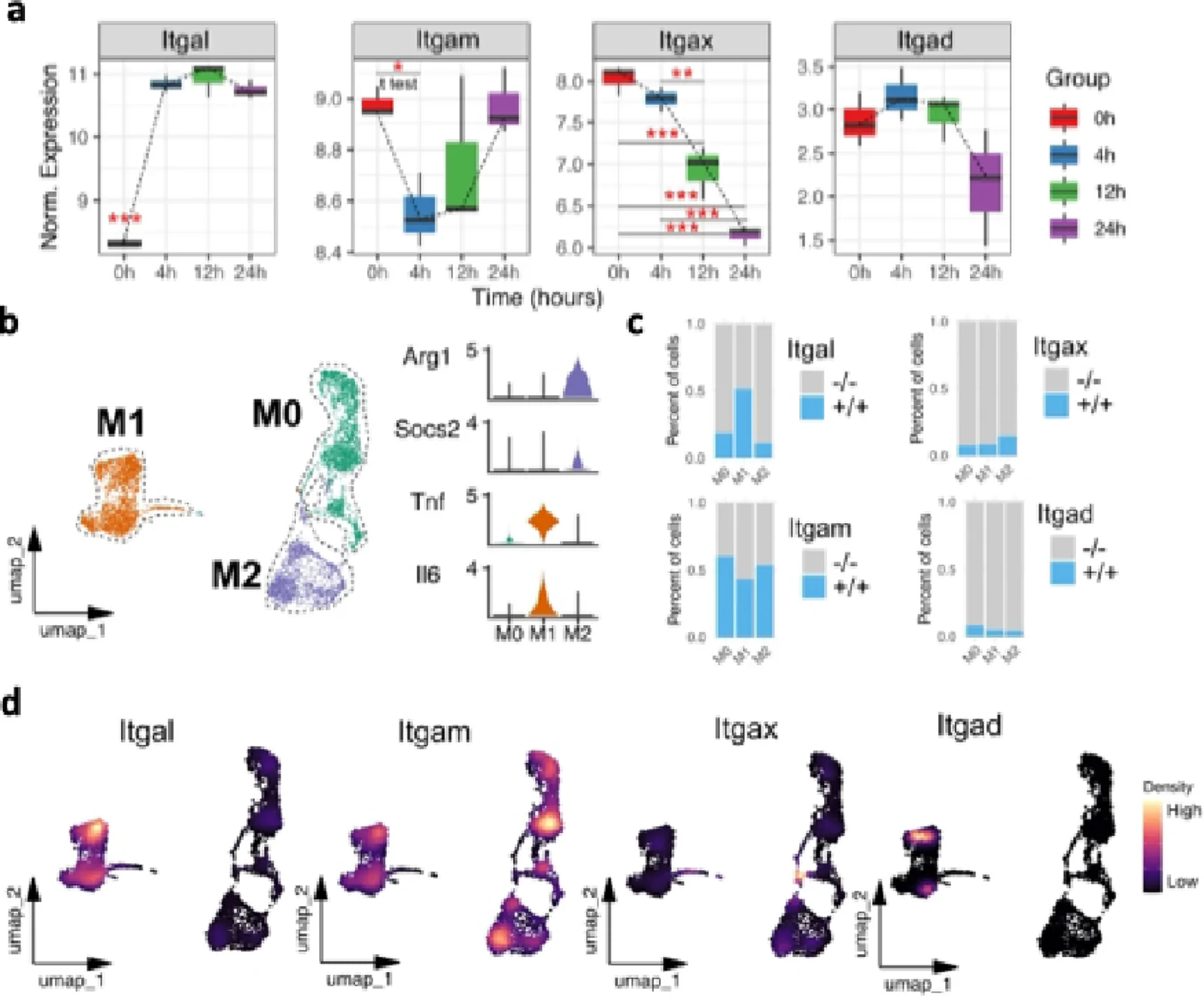
Expression patterns of β2-integrin subunits in bone marrow-derived macrophages. a) Temporal expression profiles of the β2-integrin subunits Itgal, Itgam, Itgax, and Itgad in bone marrow-derived macrophages at 0, 4, 12, and 24 h following LPS stimulation, based on publicly available bulk RNA-seq data. b) UMAP visualization of bone marrow-derived macrophages under M0 (baseline), M1 (LPS-stimulated), and M2 (IL-4-stimulated) conditions, together with the corresponding marker genes defining each macrophage state. c) Proportions of macrophages expressing Itgal, Itgam, Itgax, or Itgad across M0, M1, and M2 polarization states. d) Density plots showing the distribution and relative expression levels of Itgal, Itgam, Itgax, and Itgad across individual macrophages in the M0, M1, and M2 states.

**Figure 2:**
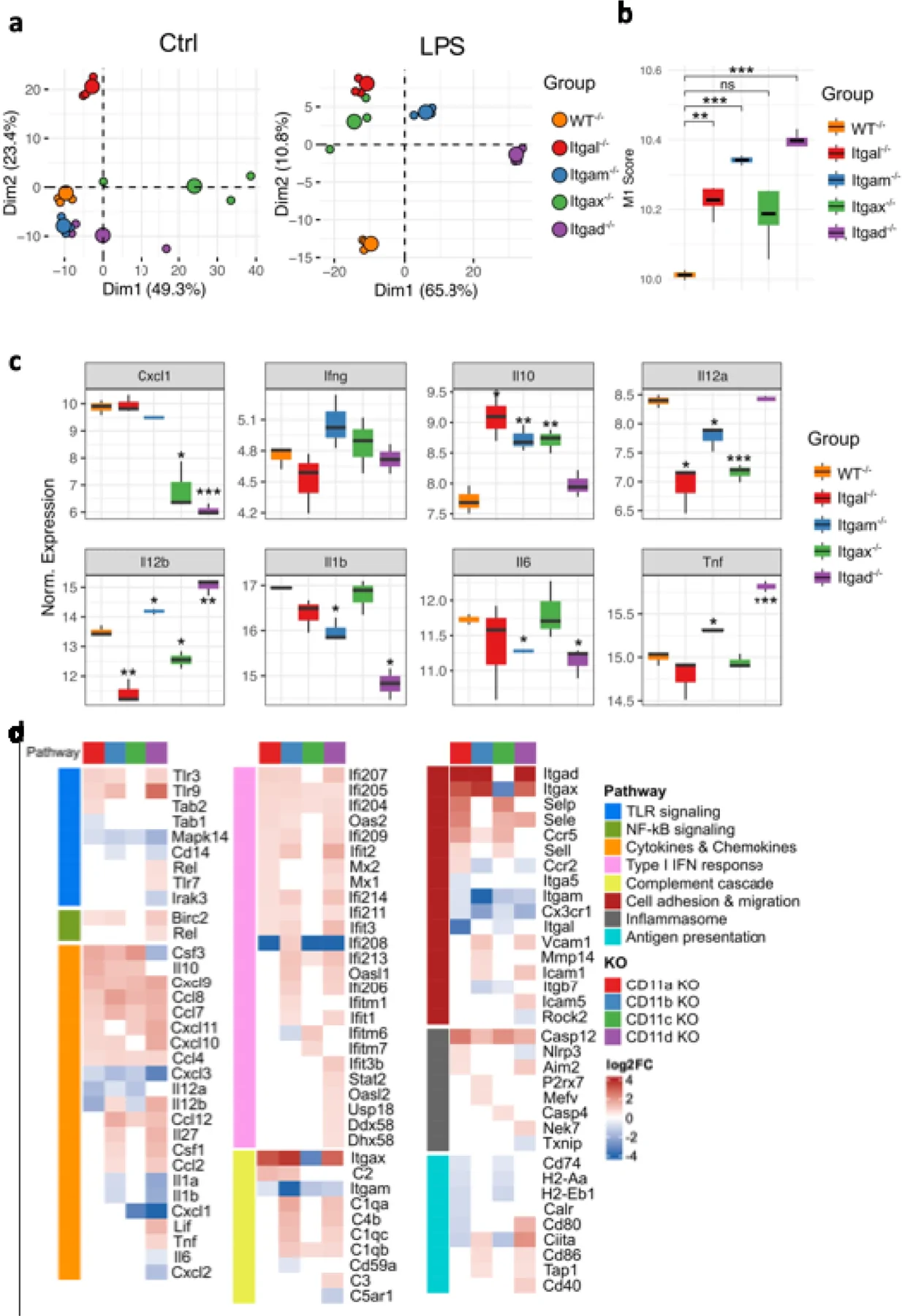
Transcriptomic analysis of β2-integrin-deficient bone marrow-derived macrophages. a) Principal component analysis (PCA) showing the transcriptional separation of WT and β2-integrin-deficient macrophages under control and LPS-stimulated conditions. b) M1 macrophage signature scores across WT, Itgal−/−, Itgam−/−, Itgax−/−, and Itgad−/− macrophages-under LPS stimulation. c) Expression profiles of inflammatory cytokines and chemokines across β2-integrin-deficient macrophages following LPS stimulation. d) Heatmaps showing the expression patterns of genes associated with major inflammatory and immune pathways, including TLR, cytokine and chemokine, type I interferon, complement, cell adhesion and migration, inflammasome, and antigen-presentation pathways, across β2-integrin-deficient macrophages following LPS stimulation.

**Figure 3:**
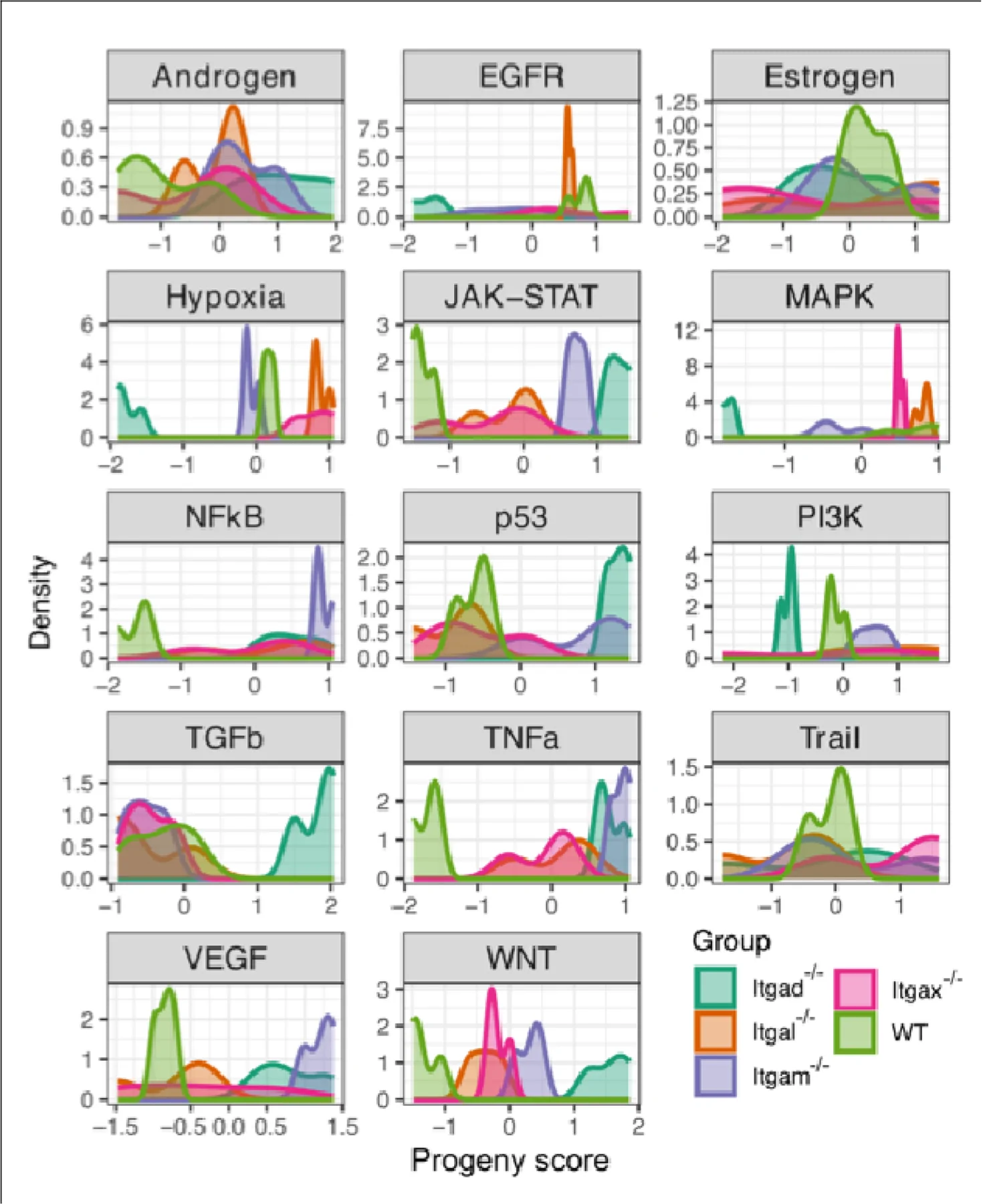
PROGENy-inferred signaling pathway activity in β2-integrin-deficient macrophages. PROGENy pathway activity scores for WT, Itgal−/−, Itgam−/−, Itgax−/−, and Itgad−/− bone marrow-derived macrophages, showing the activity profiles of major signaling pathways, including androgen, EGFR, estrogen, hypoxia, JAK–STAT, MAPK, NFκB, p53, PI3K, TGFβ, TNFα, TRAIL, VEGF, and WNT signaling.

**Figure 4:**
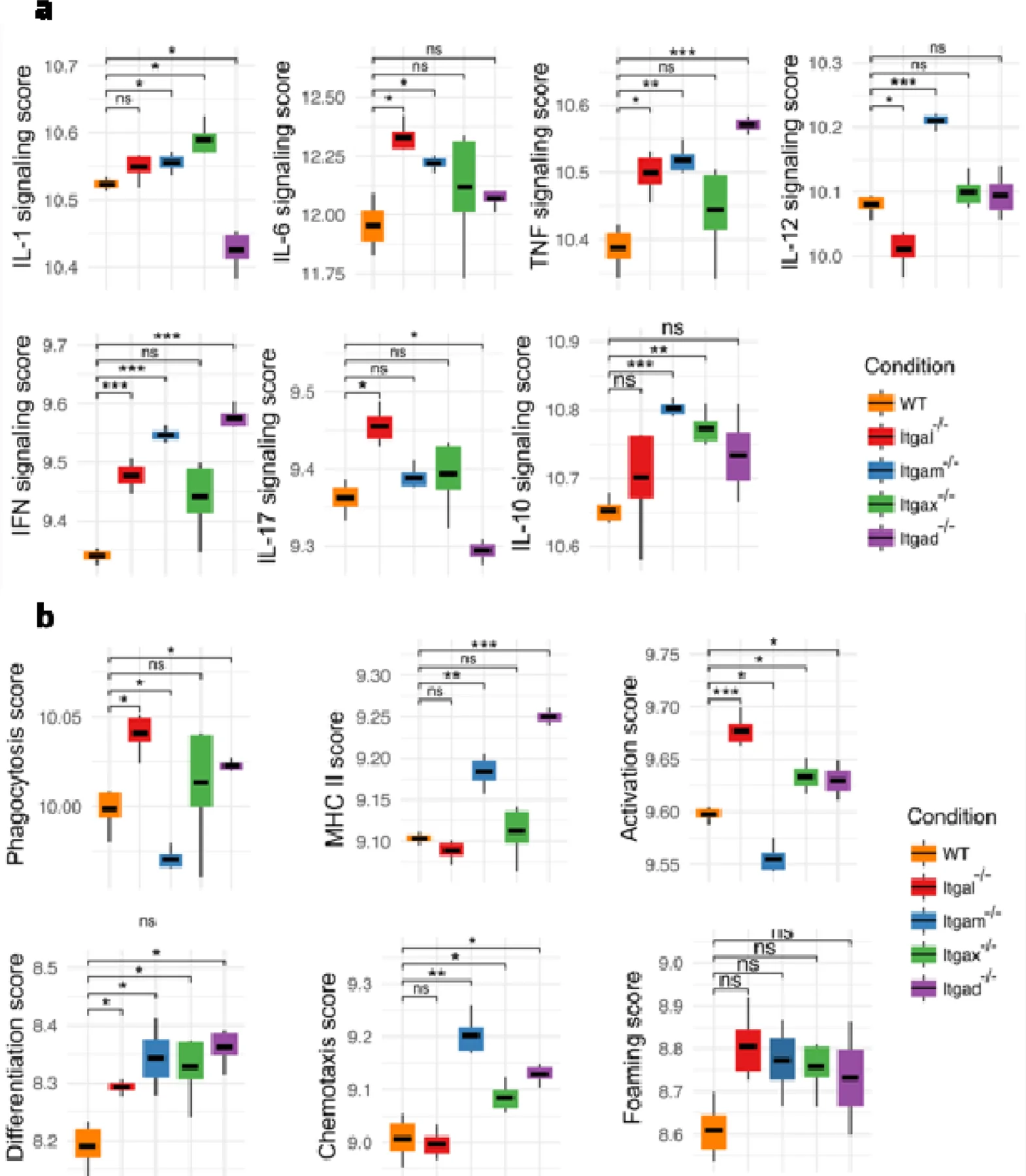
β2-integrin-dependent regulation of inflammatory signaling and macrophage functional programs. a) Module scores for inflammatory signaling programs, including IL-1, IL-6, TNF, IL-12, IFN, IL-17, and IL-10 signaling, across WT and β2-integrin-deficient bone marrow-derived macrophages. b) Module scores for macrophage functional programs, including phagocytosis, MHC class II expression, macrophage activation, differentiation, chemotaxis, and foaming, across WT and β2-integrin-deficient bone marrow-derived macrophages.

**Figure 5:**
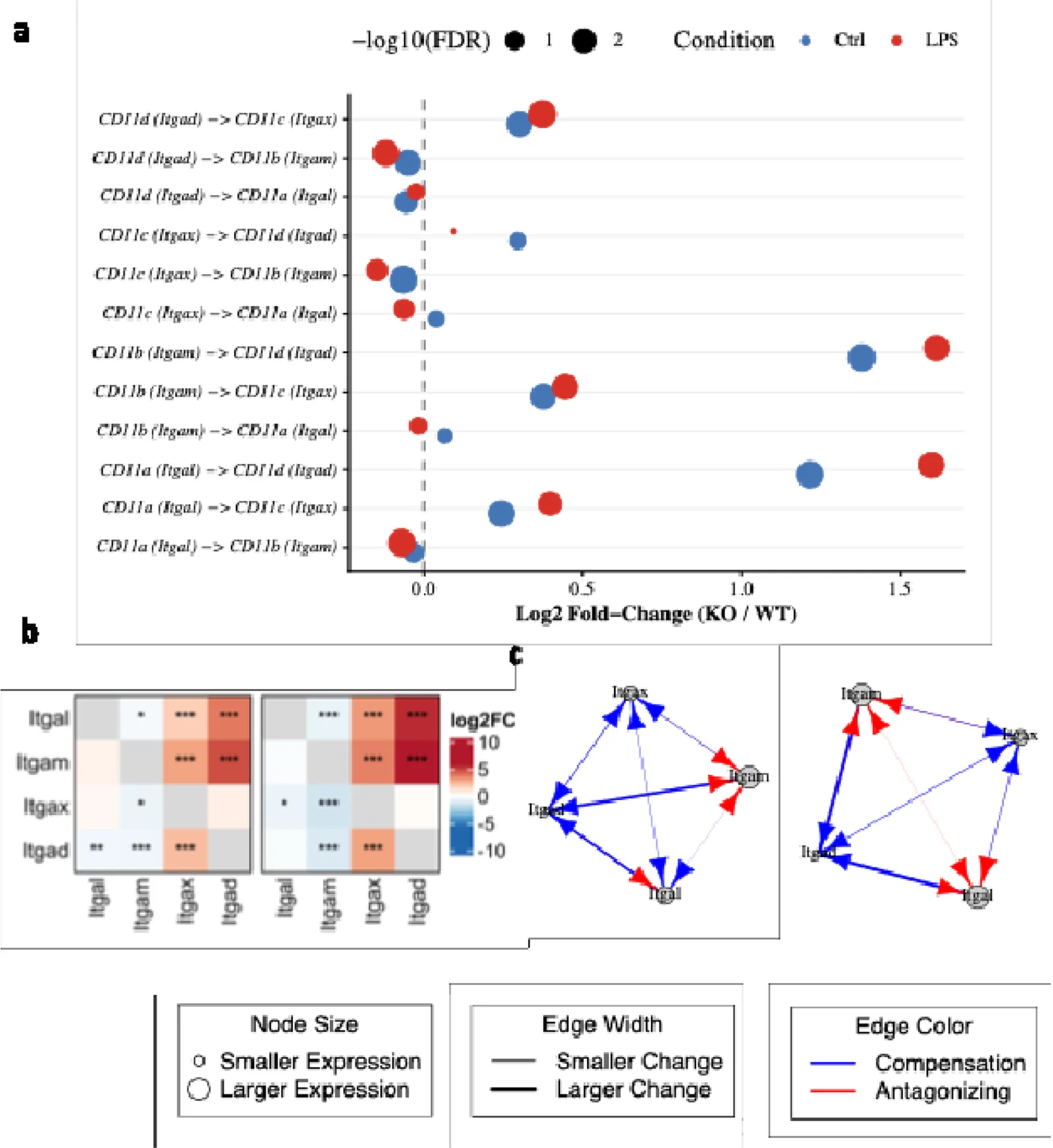
Interdependent regulatory network among β2-integrin subunits in bone marrow-derived macrophages. a) Dot plots showing the effects of individual β2-integrin subunit deletion on the expression of the remaining β2-integrin subunits under control and LPS-stimulated conditions. b) Heatmaps showing the magnitude and statistical significance of changes in β2-integrin expression following deletion of individual β2-integrin subunits under control and LPS-stimulated conditions. c) Network representation of the inferred regulatory relationships among β2-integrin subunits under control and LPS-stimulated conditions, with edge width representing the magnitude of the expression change and edge color indicating compensatory or antagonistic relationships.

**Figure 6:**
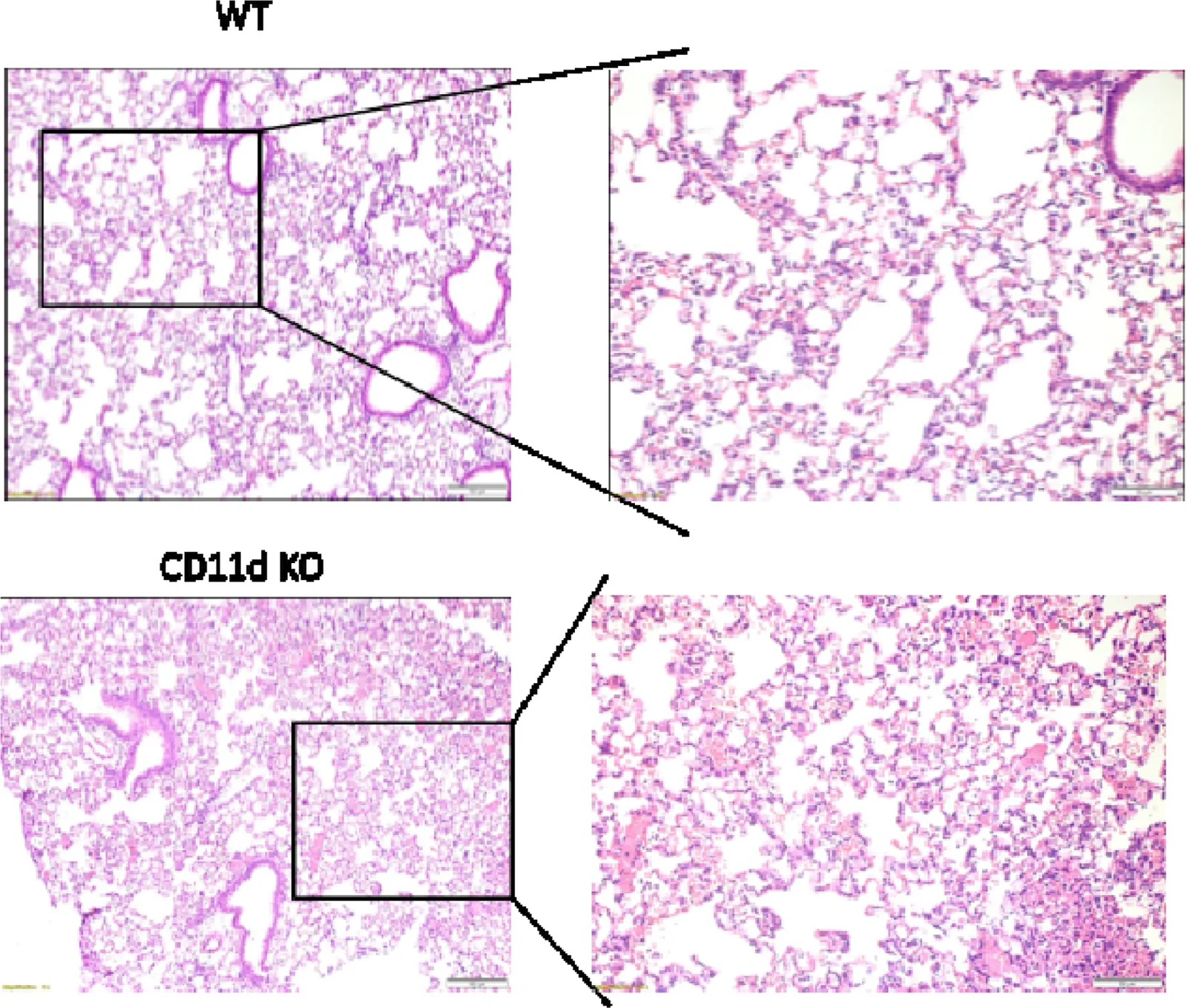
Hematoxylin and eosin (H&E) staining of lung tissue sections from wild-type (WT) (top panel) and CD11d-deficient mice (bottom panel) following intranasal LPS instillation. Lungs were obtained at 6 hours after LPS instillation. For both WT and CD11d KO mice, right panel is a blow-out image of left panel. left panel; white bar 100 μm, right panel; white bar 50 μm.

## Data Availability

Raw data generated from the bulk RNA-sequencing experiments have been deposited in the Gene Expression Omnibus (GEO) under accession number GSE345401. Publicly available bulk and single-cell RNA-sequencing datasets were retrieved from the GEO database under accession numbers GSE103958 and GSE161125, respectively.

## Code availability

Code used for every algorithm followed in data processing and analysis is fully referenced within the specific Methods sections.
